# Construction of enterovirus G expressing reporter genes for antiviral drug screening assays

**DOI:** 10.1186/s12917-025-04960-0

**Published:** 2025-08-13

**Authors:** Kaige Chen, Dalin Hong, Lingyou Zeng, Jinni Bian, Shiting Huang, Yifeng Qin, Yeshi Yin, Weijian Huang, Ying Chen, Zuzhang Wei, Kang Ouyang

**Affiliations:** 1 College of Animal Science and Technology, Guangxi University, Nanning, 530005 Guangxi China; 2 Guangxi Zhuang Autonomous Region Engineering Research Center of Veterinary Biologics, Nanning, 530005 China; 3 Guangxi Key Laboratory of Animal Reproduction, Breeding and Disease Control, Nanning, 530005 China

**Keywords:** Enterovirus G, Infectious clone, Reporter gene, Antiviral drug screening

## Abstract

The emergence of Enterovirus G strains harboring recombinant papain-like protease (EV-G-PLP) poses a significant threat to the global swine health with zoonotic potential. Addressing the critical lack of targeted treatments, we developed a reverse genetics system for the CH/20GXNN/PLP2020 strain and generated three isogenic reporter recombinants (GFP/RFP/iLOV) through precise PLP gene substitution. Viral progeny exhibited parental-like replication kinetics, with r20GXNN-iLOV demonstrating genetic stability (> 10 passages) compared to GFP/RFP variants. Plaque phenotyping revealed an inverse correlation between plaque size and insert length at the 2C/3A junction. Leveraging this system, we engineered a high-throughput screening platform identifying four anti-EV-G compounds: niclosamide (0.02 µM), salinomycin (2 µM), chloroquine phosphate (12 µM), and ribavirin (400 µM). This study establishes a reverse genetics platform enabling picornavirus research advancement and reveals structural plasticity in the 2C/3A junction that facilitates antiviral screening. Furthermore, it identifies EV-G-specific drug candidates while providing a framework for pan-picornaviral (including EV-A71) compound discovery.

**Supplementary Information:**

The online version contains supplementary material available at 10.1186/s12917-025-04960-0.

## Introduction

Enterovirus G (EV-G), part of the *Picornaviridae* family and *Enterovirus* genus, commonly infects pigs of all ages, with the most severe impact on weaned piglets [1, 2]. Symptoms of EV-G infection range from asymptomatic to skin lesions, weight loss, fever, and neurological issues. Infected piglets often show weight loss, hind limb ataxia, and circling behavior [3, 4]. Pathological studies have revealed brain congestion, neuronal degeneration, necrosis, and vacuolation of Nissl bodies in affected piglets [4].

The EV-G genome comprises a single positive-strand RNA with a poly A tail, an open reading frame (ORF), and 5’ and 3’ untranslated regions (UTRs) [5, 6]. The ORF encodes four structural proteins (VP4, VP2, VP3, VP1) and seven non-structural proteins (2A, 2B, 2C, 3A, 3B, 3C, 3D) [7].

EV-G was first isolated from pig skin tissue in 1972 [8]. Since then, outbreaks have occurred in many countries, including the United States, China, and Japan [6, 9–14]. Our previous seroepidemiological survey conducted in Guangxi, China, from 2019 to 2021, revealed that 68.78% of serum samples and all pig farm samples were positive for EV-G, showing high antibody positivity across all age groups [15].

Recently, natural recombination events between the EV-G genome and the PLP gene of Porcine Torovirus (PtoV) have been reported. In 2017, a recombinant EV-G strain with the PLP gene (EV-G-PLP) was identified in Texas, USA [16]. Subsequently, similar strains were detected in Belgium and China, with the PLP gene substituting part of the EV-G genome [17, 18]. This pattern has also been observed in Japan and persists in pig farms [6, 19]. Currently, EV-G-PLP recombinant strains have been identified in an increasing number of countries and regions. However, the mechanisms of recombination, changes in pathogenicity, and associated antiviral drugs have not been revealed, highlighting an urgent need for further research.

Infectious clones are crucial for studying RNA virus gene structure, function, replication, infection, and for developing vaccines [20]. Previous reports have constructed infectious clones of EV-G-PLP strains using various vectors and promoter sequences [21]. Our earlier work also demonstrated the feasibility of constructing an infectious clone of the EV-G virus, supporting the potential for creating an infectious clone of the EV-G-PLP virus [22]. This established a foundation for further research into the pathogenic mechanisms of PLP, the screening of anti-EV-G drugs, and the potential use of EV-G as a viral vector for accommodating exogenous gene fragments in vaccine development.

## Materials and methods

### Cells, viruses, plasmids and antibodies

Marc-145 cells were cultured in Dulbecco’s modified Eagle’s medium (DMEM) with 10% fetal bovine serum (FBS), 100U/mL penicillin, and 100 µg/mL streptomycin. The EV-G-PLP strain, CH/20GXNN/2020/PLP, was isolated from porcine diarrhea samples in Nanning, Guangxi, and stored at −80 °C. The pBluescript II SK plasmid is stored in our laboratory. Polyclonal antibodies against VP1 (anti-EV-G-VP1 PcAb) and PLP (anti-EV-G-PLP PcAb) were generated in a previous study and are currently maintained in our laboratory.

### RNA extraction and RT-PCR

Total RNA was harvested from the culture supernatants of EV-G CH/20GXNN/2020/PLP-infected cells by using a Viral DNA/RNA Miniprep Kit (Axygen Scientific, Union City, USA). Oligo dT primer was used to synthesize cDNA from the RNA. The cDNA served as a template for PCR amplification of the viral genome fragments.

### Construction of the full-length infectious clone of EV-G-PLP

**Fig. 1:**
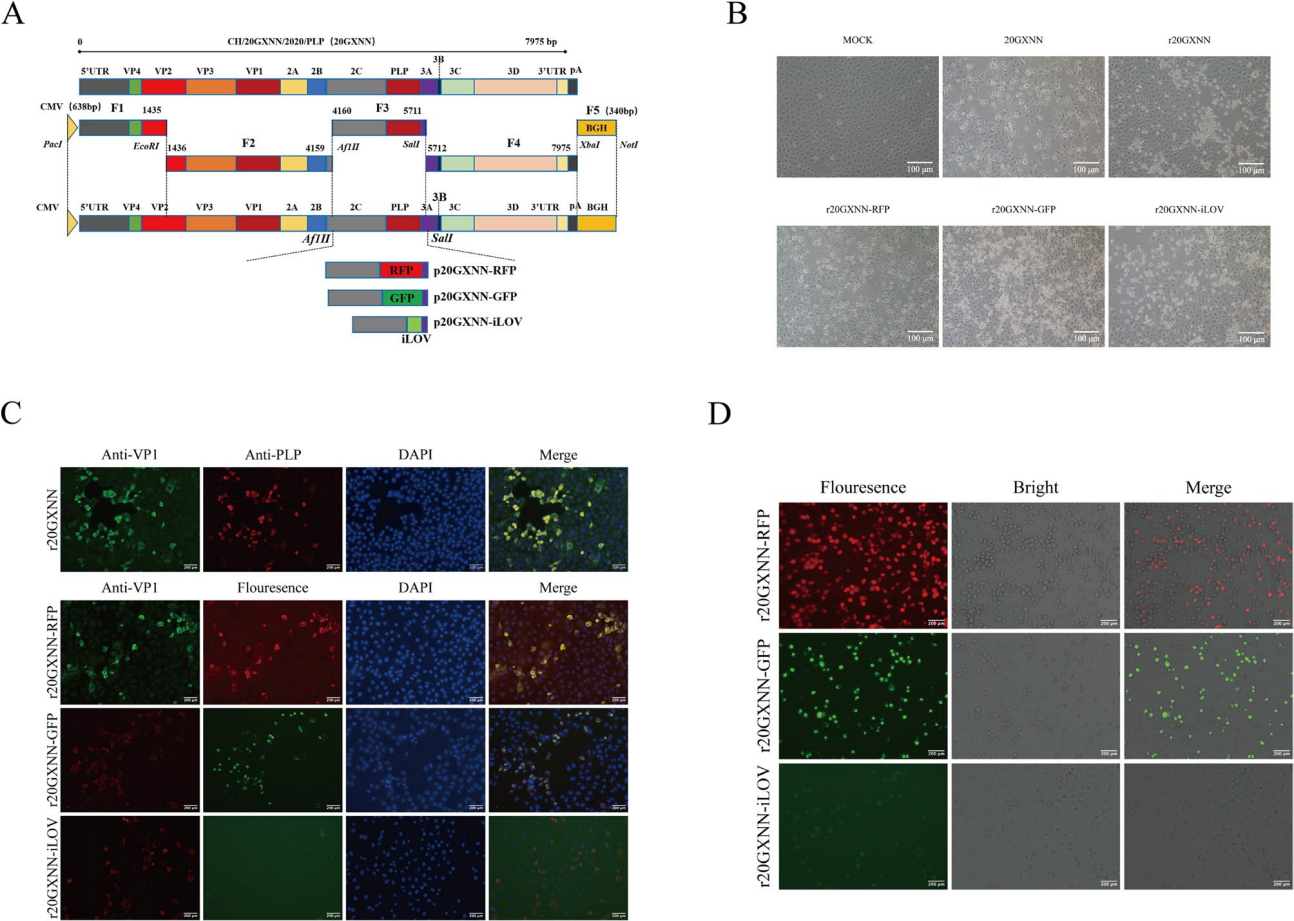
Construction and characterization of the recombinant reporter virus. **A** Schematic of the recombinant reporter viruses infectious clone. The 20GXNN genome was cloned into a vector in five fragments (F1–F5). The PLP gene was replaced with a reporter gene using overlap extension PCR. **B** Cytopathic effects in Marc-145 cells infected with rescued viruses and 20GXNN. **C** IFA analysis of viral VP1 and PLP protein expression in Marc-145 cells infected with P2 generations of the rescued viruses, respectively. **D** The fluorescence expression in Marc-145 cells infected with recombinant reporter viruses was analyzed. Marc-145 cells were infected with recombinant reporter viruses at an MOI of 0.01, and the expression of RFP, GFP, or iLOV was observed under a fluorescent microscope following infection

The strategy for constructing the full-length infectious clone of EV-G CH/20GXNN/2020/PLP is shown in Fig. 1A. Primers were designed with restriction sites (*Pac* I, *Not* I, *Eco R* I, *Afl* II, *Sal* I, and *Xba* I) (Table 1). The genome’s frontend was fused with the CMV promoter into fragment I (F1) and cloned into the plasmid using *Pac* I and *Not* I. Fragments II-IV (F2-4) were cloned using *Eco R* I, *Afl* II, *Sal* I, and *Not* I. Poly (A) was added at the genome’s end with primer R4, and the polyadenylation signal (BGH) was added using *Xba* I and *Not* I, forming plasmid p20GXNN.

**Table 1:** Summary of Seroprevalence against EV-G in swine from 2019 to 2021

Primer name	Primer sequence (5’−3’)	Purpose
CMF-F	CGACGGCCAGTGAGGGCCGGCCATTTAAA*TTAATTAA*GGACATTGA	Amplification of CMV gene
CMV-R	GAACAACCCACAGGCTGTTTTAAGACGGTTTATATAACGAGCTCTGC	
F1	GCAGAGCTCGTTATATAAACCGTCTTAAAACAGCCTGTGGGTTGTTC	Amplification of F1 gene
R1	TCCACCGCGGTG*GCGGCCGC*ATTGCC*GAATTC*CGGTTGG	
F2	CCACCCGCCAACCG*GAATTC*GGCAATGTCATGCCAGGAAAAACC	Amplification of F2 gene
R2	CCACCCGCCAACCG*GAATTC*GGCAATGTCATGCCAGGAAAAACC	
F3	ATGAAATTTATTGATTGG*CTTAAG*AACCACCTTGTGCCCCAAGCTAAGG	Amplification of F3 gene
R3	TCCACCGCGGTG*GCGGCCGC*TTCTTCTGA*GTCGAC*GCTGGCAA	
F4	ATTGCAGATCTCCTTGCCAGC*GTCGAC*TCAGAAGAAGTTAGAGAATA	Amplification of F4 gene
R4	ACCGCGGTG*GCGGCCGCTCTAGA*TTTTTTTTTTTTTTTTTTTTTTTGG ATTCAATTTTACAC	
BGH-F	TAGTC*TCTAGA*GGGTCGGCATGGCATCTCCACCTC	Amplification of BGH gene
BGH-R	ACCGCGGT*GGCGGCCGC*CATAGAGCCCACCGCATCCCCAGCA	
2C-R	TGGCTTAAATACTGGGGGTCCCTGGAATAGTGCCTCAATA	PLP genes were replaced with reporter genes by overlapping extended PCR
RFP-F	CAGGGACCCCCAGTATTTAAGCCAATGGTGAGCAAGGGCGAGGA	
RFP-R	GGGTCCTTGAAATTCTGCAGGGCGGTTTCCGGACTTGTACAGCTCG	
GFP-F	CCAGTATTTAAGAGGCGCGCCATGCCCGCCATGAAGATCG	
GFP-R	CCTTGAAATTCTGCCCCGGGGGCGAATGCGATCGGGG	
iLOV-F	CAGTATTTAAGAGGCGCGCCTTCGAAATGATAGAGAAGAAT	
iLOV-R	TCCTTGAAATTCTGCCCCGGGTACATGATCACTTCCATCGAGC	
3A-F	CGCCCTGCAGAATTTCAAGGACCCCCAACATTTAAGCCTT	
R4-1	CCACTAAGTTGTAGGCGTCAGCAACCTGAGTTTCAACTCCGTCT	
EV-G-F	CAAGCACTTCTGTYTCCCCGG	Identification of genetic marker
EV-G-R	GTTAGGATTAGCMGCATTCA	
EV-G PLP F	GTACCMTACCAYGTDCCHATGGC	Identification of stability of inserted gene in 2C/3A region
EV-G PLP R	GCAATNGCTGGDGGDGCTGG	

### Construction of recombinant infectious clones with reporter genes

Two 3Cpro cleavage sites flanking the inserted PLP gene were positioned at junction of 2C/3A junction region of EV-G-PLP strains. During PLP gene replacement, the native 3Cpro cleavage sites flanking the insertion point– ALFQGPPVFK (N-terminal) and AEFQGPPTFK (C-terminal)– were preserved to ensure proper reporter genes release. Reporter genes (RFP, GFP, and iLOV) were inserted between the 2C/3A junction region of the p20GXNN clone using overlap extension PCR(SOE-PCR), resulting in the recombinant constructs p20GXNN-RFP, p20GXNN-GFP, and p20GXNN-iLOV, respectively. The resultant infectious clones were finally verified by Sanger sequencing using EV-G PLP F/R primers at Sangon Biotech (Shanghai, China). The primers used are listed in Table 1.

### Rescue of viruses from infectious clones

To produce progeny viruses, marc-145 cells were cultured in 6-well plates and brought to 70% confluence for transfection with full-length cDNA infectious clones. The cDNAs (2 µg) from each virus were transfected into Marc-145 cells in six-well plates using Lipofectamine 2000 (Invitrogen, USA) according to the manufacturer’s instructions. At 48 h post transfection (hpi), the cultures were frozen and thawed three times, and cell debris was removed by centrifugation. The cleared supernatants (P0) were collected and used to inoculate Marc-145 cells for blind passaging until clear signs of cytopathic effects (CPEs) were observed. Rescued recombinant virus carrying reporter genes was designated as r20GXNN-GFP, r20GXNN0-RFP and r20GXNN-iLOV, aliquoted, and stored at − 80 °C for subsequent experiments.

### Indirect immunofluorescence assay (IFA)

Marc-145 cells grown in 6-well plates were infected with rescued viruses at a multiplicity of infection (MOI) of 0.01. When CPEs were observed, the cells were fixed with cold methanol for 30 min at −20°C. After blocking with 1% BSA (bovine serum albumin fraction V; Roche, Mannheim, Germany) for 1 hour at 37 °C, Marc-145 cells were incubated at 37 °C with rabbit anti-EV-G VP1 or mouse anti-EV-G PLP antibodies (1:500 dilution) for 2 hours. After washing three times with Phosphate Buffered Saline (PBS), the cells were incubated with the Alexa Fluor^®^ 488 Goat anti-Rabbit IgG (H + L) (1:2000 dilution), or Cy3-conjugated Goat Anti-Rabbit IgG (H + L) (1:500 dilution), or Cy3-conjugated Goat Anti-Mouse IgG (H + L) (1:500 dilution) for 1 h at 37°C. The cell nucleus was stained with DAPI (4’, 6-diamidino-2-phenylindole; Servicebio, Wuhan, China) for 10 min at room temperature, and fluorescent signals were visualized with a fluorescence microscope.

### Plaque assay

Marc-145 cells were grown in 6-well plates and infected with 10-fold serially diluted viral inoculates. The plates were gently shaken every 15 min and incubated at 37 °C for 1 h. After incubation, the cells were washed twice with PBS and then loaded with 2×DMEM mixed in equal volumes 1% agarose (Oxoid, Basingstoke, UK). After the gel overlay solidified, the plates were inverted and placed in an incubator at 37 °C with 5% CO_2_ for an additional 3 days. To visualize the infected cells, the cells were fixed with 10% formaldehyde and stained with 1% crystal violet solution. Subsequently, the plates were rinsed with running water and photographs were captured. The plaque diameters were measured using a ruler with ImageJ 1.8.0 software.

### Viral replication kinetics

Recombinant virus-infected Marc-145 cells were used to characterize the in vitro growth properties. Marc-145 cells were inoculated with recombinant viruses at an MOI of 0.01. Cell culture supernatants were harvested at 6, 12, 24, 36, 48, 60, 72 and 84 hpi, and the viruses were titrated using plaque assays. Each sample was run in triplicate to determine accuracy.

### Genetic stability of viruses carrying reporter genes

The stability of the rescued recombinant virus was determined by PCR and sequencing. RNA was extracted from Marc-145 cells infected with the 1 st, 3rd, 5th, 7th, and 10th generation recombinant viruses. RNA was then reverse-transcribed into cDNA, which was used as a template for PCR to amplify and detect the 2C/3A region using specific primers (Table 1). The obtained PCR amplification fragment was sequenced to further identify the reporter gene in the rescued virus.

### Cell viability assay

Cell viability was measured using Cell Counting Kit-8 (CCK-8, APE×BIO) according to the manufacturer’s protocol. Marc-145 cells in 96-well plates were treated with indicated doses of drugs, followed by incubation at 37 °C with 5% CO₂ for 36 h. Then, the CCK-8 solution was added, and the cells were incubated at 37 °C for 45 min under light-restricted conditions. Absorbance was measured at 450 nm using a microplate reader (Tecan, M200 PRO, CH). Cell viability was calculated using the formula: cell viability (%) = [OD_450_(compound)- OD_450_(blank)]/[OD_450_(control)-OD_450_(blank)] × 100%.

### Antiviral testing

Six compounds were used in this study to explore their potential as antiviral drugs: ribavirin, salinomycin, niclosamide, chloroquine phosphate, Melaleuca alternifolia essential oil, and chitosan. Marc-145 cells were seeded into a 12-well plate for 24 h and infected with r20GXNN-GFP at an MOI of 0.1 for 1 h. The infected cells were then incubated for 36 h either in the absence of the compound or at indicated concentrations within its maximum safe solubility range. Antiviral activity was evaluated through plaque assays on culture supernatants. Images were captured using an Olympus inverted fluorescence microscope, and subsequent quantitative analysis of the fluorescence area in the acquired images was performed utilizing Image J software.

### Statistical analyses

All experiments in this study were performed independently at least twice or included multiple biological replicates, with representative results presented. Statistical analyses were conducted using GraphPad Prism 6 software (GraphPad Software, CA, USA), employing one-way analysis of variance (ANOVA), with a P-value of < 0.05 considered statistically significant. Nucleotide and protein sequence analyses were conducted with SnapGene 6 software (GSL Biotech LLC, USA).

## Results

### Construction of recombinant EV-G variants infectious clones carrying reporter genes

To construct an infectious clone of the EV-G-PLP virus, the viral genome, along with the CMV promoter, poly(A) tail, and BGH signal sequence at both ends, was divided into five segments and cloned into the vector, resulting in the construction of p20GXNN (Fig. 1A). Sequencing confirmed that the amino acid sequences of the rescued virus and the original strain were identical. To construct recombinant infectious EV-G variants carrying different reporter genes, the infectious cDNA clone p20GXNN was used as a template. Using the overlap extension PCR method, the PLP gene in fragment III of p20GXNN was replaced with reporter genes (RFP, GFP, iLOV). Fragment III carrying the reporter gene was then substituted into p20GXNN, resulting in p20GXNN-RFP, p20GXNN-GFP, and p20GXNN-iLOV constructs. These constructs were verified by sequencing.

### Rescue and characterization of recombinant virus carrying reporter genes

The recombinant plasmids were transfected into Marc-145 cells. After 48 h, cells were collected as passage 0(P0) generation samples and passaged in Marc-145 cells. Marc-145 cells transfected with p20GXNN, p20GXNN-GFP, and p20GXNN-iLOV plasmids observed typical CPE at 20 h post transfection (hpi) in the P1 generation, while those transfected with p20GXNN-RFP plasmid observed typical CPE at 18 hpi in the P2 generation (Fig. 1B). The rescued viruses were named r20GXNN, r20GXNN-RFP, r20GXNN-GFP, and r20GXNN-iLOV, respectively. In order to verify the intracellular expression of the EV-G protein, Marc-145 cells at 18 hpi with recombinant viruses were subjected to an IFA test. As shown in Fig. 1C, VP1 and PLP proteins were expressed following r20GXNN infection. Furthermore, VP1 expression was detected in the rescued viruses in which the PLP gene was substituted with reporter genes. No viral protein expression was observed in the mock-infected control group. At 18 hpi of the P2 generation virus, fluorescent positive cells were visible under a fluorescence microscope, confirming reporter gene expression in cells infected with reporter gene-carrying viruses (Fig. 1D). Compared to r20GXNN-iLOV, more intense fluorescence was observed following infection with r20GXNN-RFP and r20GXNN-GFP.

### Viral replication kinetics and plaque sizes of viruses carrying reporter genes

**Fig. 2:**
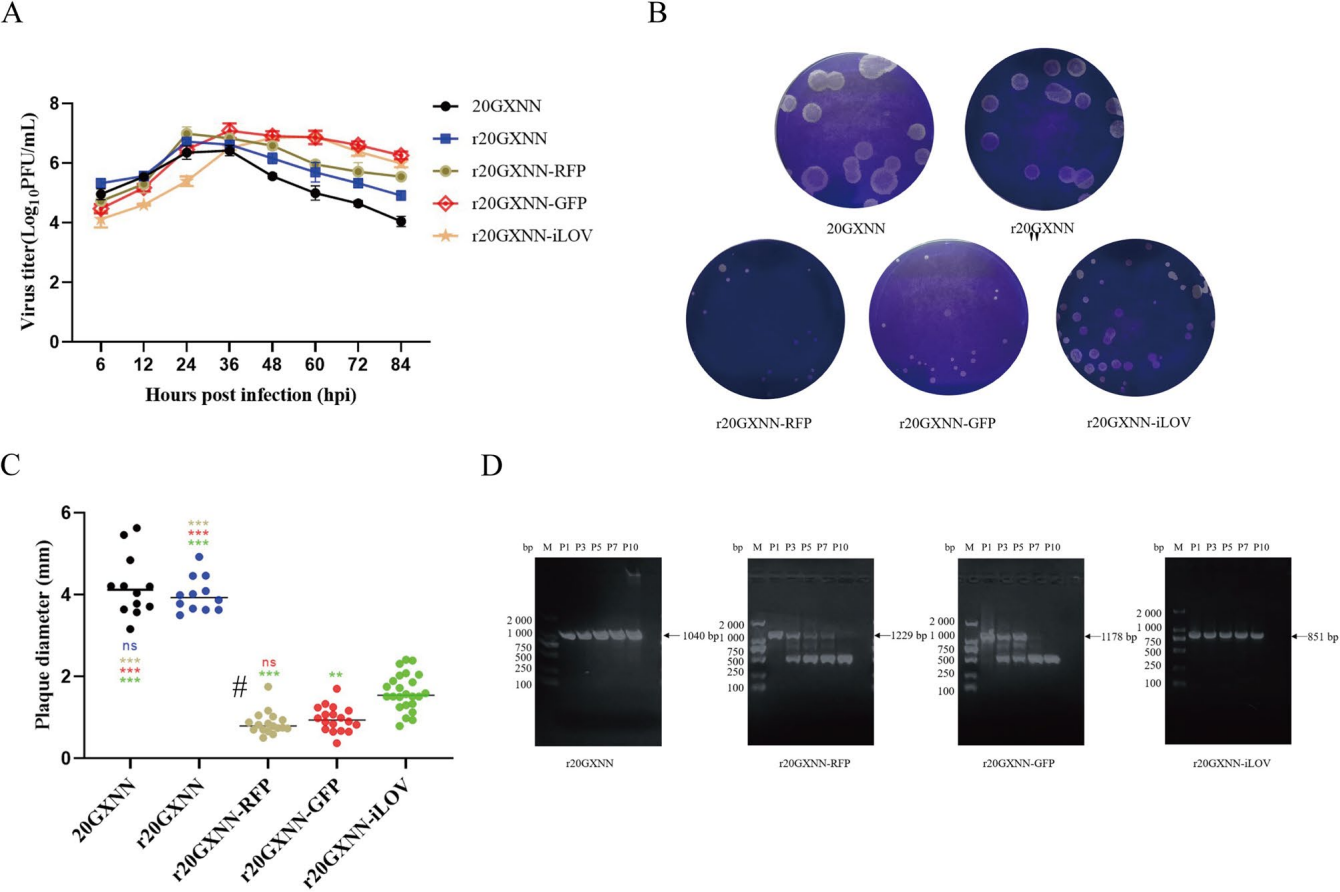
The growth characteristics and genetic stability analysis of the rescued viruses. **A** Growth curves of rescued viruses and 20GXNN in Marc-145 cells. Marc-145 cells were infected with rescued viruses and 20GXNN at an MOI of 0.01. Supernatants were collected and titrated using plaque assays at the indicated time points. **B** Plaque morphology of rescued viruses and 20GXNN in Marc-145 cells. **C** The measurement of plaque diameters and the analysis of significant differences are depicted. The horizontal lines represent the average values of the measured plaque diameters. Asterisks indicate the levels of statistical significance: * for *p* < 0.05, ** for *p* < 0.01, and *** for *p* < 0.001. **D** Genetic stability of the recombinant reporter viruses and r20GXNN. Agarose gel images showing the DNA fragments encompassing the PLP gene and reporter gene insertion region, amplified by RT-PCR using RNA extracted from cells infected with passages 1, 3, 5, 7, and 10 of the viruses

Marc-145 cells were infected with each rescued virus and the parental virus at an MOI of 0.01. Samples collected at various time points were used to determine viral titers and plot the multi-step growth curve (Fig. 2A). The growth trends of the rescued strains were similar to the parental strain 20GXNN, with detectable viruses at 6 hours. 20GXNN, r20GXNN, and r20GXNN-RFP reached peak replication at 24 h with titers between 2.7 and 9.7 × 10^6^ PFU/mL, while r20GXNN-△PLP and r20GXNN-GFP peaked at 36 h with titers of 3.2 × 10^6^ to 1.25 × 10^7^ PFU/mL. Plaque assays showed similar plaque diameters for r20GXNN and 20GXNN (~ 4 mm), with no statistical difference (Fig. 2B and C). The reporter gene-carrying strains produced smaller plaques: r20GXNN-RFP (0.86 mm), r20GXNN-GFP (0.96 mm), and r20GXNN-iLOV (1.62 mm).

### Genetic stability of rescued viruses with inserted reporter genes

To monitor the genetic stability of recombinant viruses, the viruses were subjected to ten consecutive passages in Marc-145 cells. Viral RNA was extracted from passages 1, 3, 5, 7, and 10, and analyzed by RT-PCR using specific primers to examine the presence of reporter genes inserted in the 2C/3A region (Fig. 2d). The PLP gene in r20GXNN and the iLOV gene in r20GXNN-iLOV remained stable across 10 passages. However, r20GXNN-GFP and r20GXNN-RFP exhibited reporter gene loss starting from passage 3, as indicated by decreasing fluorescence in subsequent passages.

### r20GXNN-GFP as a tool for antiviral drug screening

**Fig. 3:**
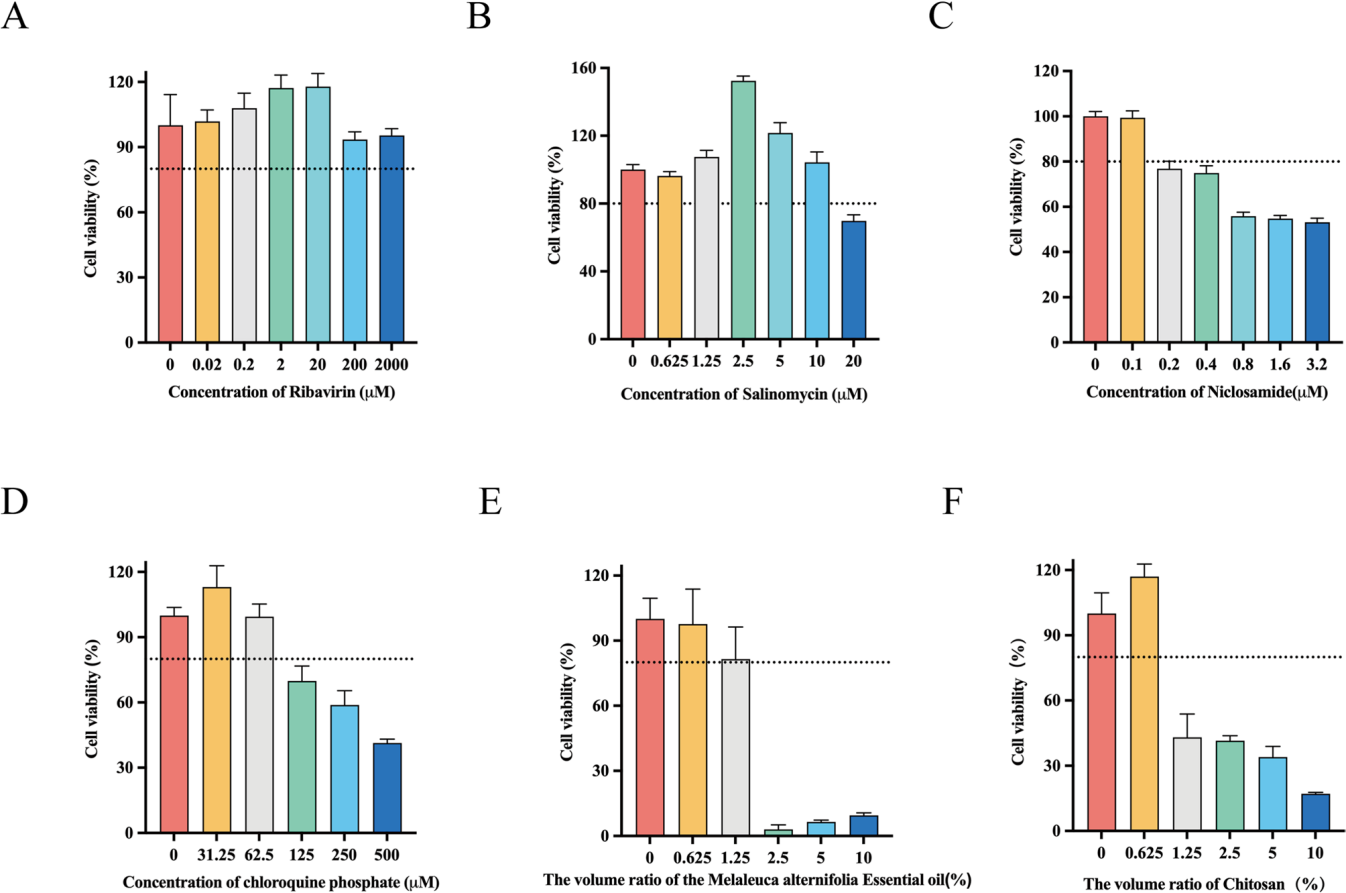
Safe concentrations of drugs for Marc-145 cells. A-F Marc-145 cells were treated with ribavirin (0, 0.02, 0.2, 2, 20, 200 and 2000 µM), Salinomycin (0, 0.625, 1.25, 2.5, 5, 10 and 20 µM), Niclosamide (0, 0.1, 0.2, 0.4, 0.8, 1.6 and 3.2 µM), Chloroquine phosphate (0, 31.25, 62.5, 125, 250 and 500 µM), Melaleuca alternifolia Essential oil (0, 0.625, 1.25, 2.5, 5 and 10 %) and Chitosan (0, 0.625, 1.25, 2.5, 5 and 10 %) for 24 h. At the end of the incubation, cell viability was measured using the CCK-8 Assay

r20GXNN-GFP, exhibiting strongest fluorescence, was selected to validate the suitability of the reporter viruses for antiviral screening. The cytotoxic effects of six compounds—ribavirin, salinomycin, niclosamide, chloroquine phosphate, melaleuca alternifolia essential oil, and chitosan—were evaluated using cell viability assays in Marc-145 cells. The maximal non-toxic dose (MNTD values), corresponding to an 80% cell survival rate, was used as the maximal drug concentration for subsequent in vitro antiviral drug screening experiments. As shown in Fig. 3, the safe concentrations in Marc-145 cells for ribavirin, salinomycin, niclosamide, chloroquine phosphate, melaleuca alternifolia essential oil, and chitosan were 2000 µM, 10 µM, 0.1 µM, 62.5 µM, 1.25%, and 0.625%, respectively.

**Fig. 4:**
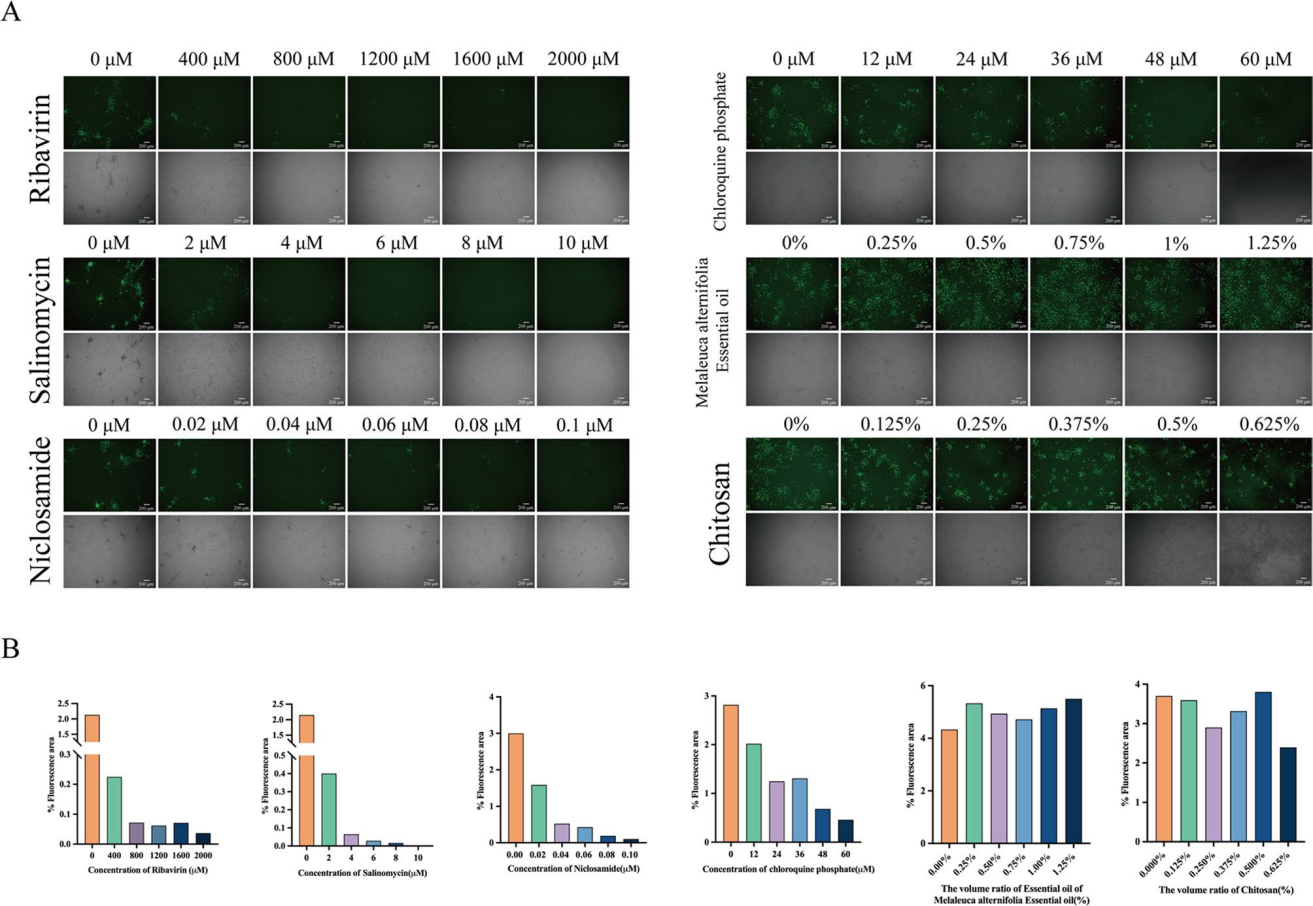
Fluorescence analysis of drug antiviral activity. **A** Marc-145 cells were infected with r20GXNN-GFP at an MOI of 0.1 and incubated with culture media containing indicated drug concentrations. At 24 hpi, GFP expression was observed under a fluorescent microscope to assess viral proliferation inhibition. **B** Fluorescence images were analyzed using Image J software, with the image type converted to 8-bit format, and the fluorescence area was quantified by applying the Renyientropy thresholding method

**Fig. 5:**
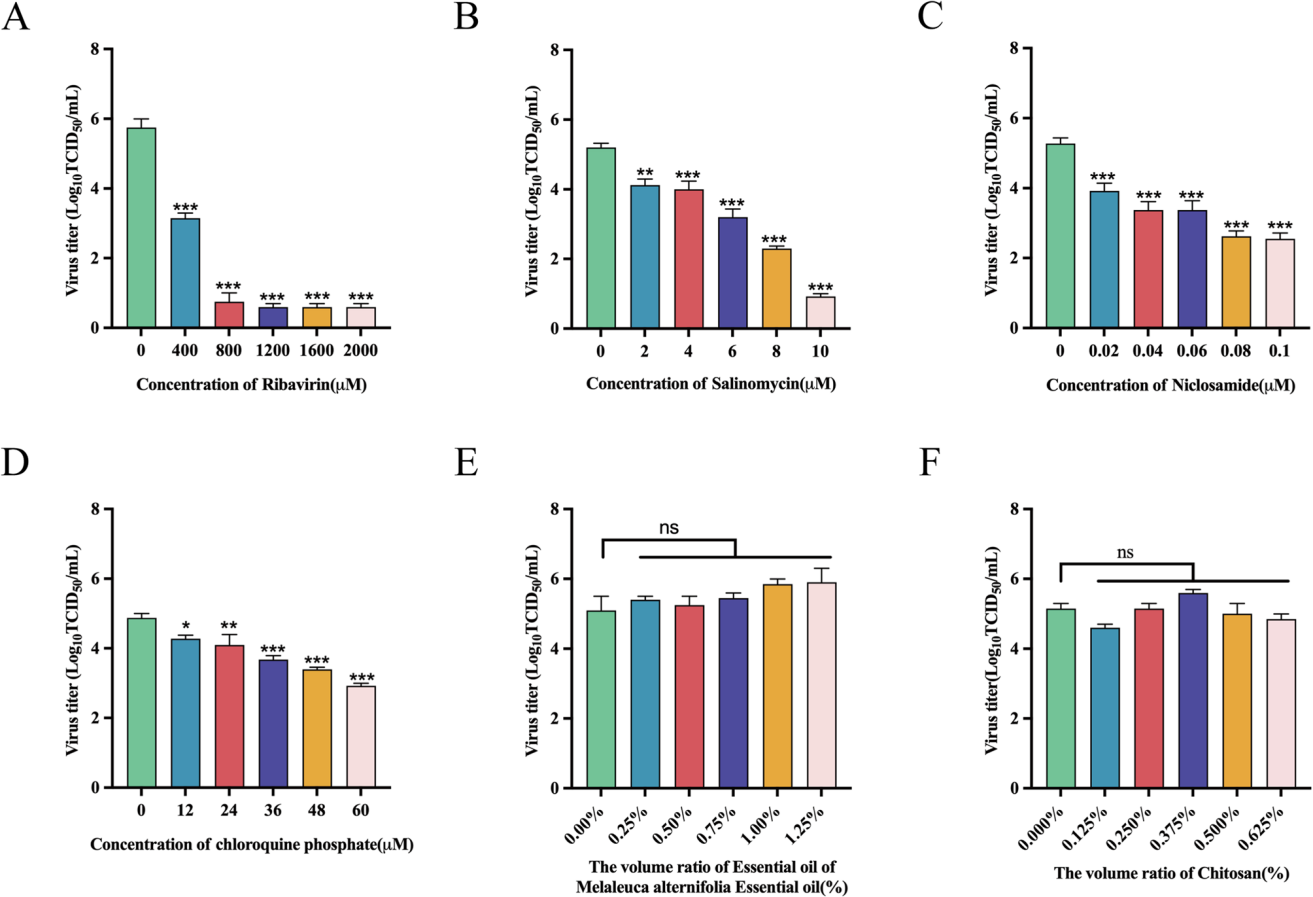
Drug-treated sample titration. A-F At 24 hpi, supernatants from r20GXNN-GFP-infected cells treated with designated drug concentrations were collected, and virus titers were determined by TCID50. Error bars represent the standard error from three independent experiments. Drug-treated groups were compared to the drug-free control group: * for p < 0.05, ** for p < 0.01, and *** for p < 0.001.

Within the safe concentration range, r20GXNN-GFP was inoculated into Marc-145 cells at an MOI of 0.1 and incubated at 37 °C for 1 h. Subsequently the cells were treated with the test compounds to determine their inhibitory effects against viral replication post-infection. After 24 h of incubation with test compounds, the cell monolayers were observed in a randomly selected fluorescence microscope field of view and culture supernatants were harvested to determine the production levels of extracellular infectious virions. Ribavirin, salinomycin, niclosamide, and chloroquine phosphate demonstrated a reduction in fluorescence with increasing drug concentrations, with ribavirin and salinomycin exhibiting the most pronounced effects. In contrast, Melaleuca alternifolia essential oil and chitosan showed no significant changes in fluorescence (Fig. 4A and B). Virus titers, determined by the TCID_50_ method, supported these observations. Inhibitory effects were observed for ribavirin, salinomycin, niclosamide, and chloroquine phosphate at concentrations of 400 µM, 2 µM, 0.02 µM, and 12 µM, respectively (Fig. 5).

## Discussion

Enterovirus are ubiquitous worldwide and are transmitted mainly through the fecal–oral and respiratory routes following a seasonal pattern [23]. Recent reports have revealed natural recombination between EV-G and the PLP gene of porcine torovirus, a rare interspecies viral event that may result in a broader host range, heightened virulence, and increased potential for immune evasion [24, 25]. The PLP gene, with its DUB and deISGylating activities, contributes to viral evasion of the host immune response. The PLP of EV-G, the Lpro of the foot-and-mouth disease virus (FMDV), and the PLP of coronaviruses all inhibit host antiviral responses through distinct mechanisms, thereby facilitating viral replication [21, 26, 27]. Studies on inhibitors targeting coronavirus PLpro, a member of the PLP family of enzymes, demonstrate that blocking its deubiquitinating (DUB) and deISGylating activities can suppress viral immune evasion and replication [28]. These findings suggest that broad-spectrum PLP inhibitors may address evolutionary threats posed by multiple viruses, including EV-G, though further validation of their applicability across diverse viral species is required.

No drug or vaccine has been approved for EV-G infection and therefore, much effort is still needed to elucidate the pathogenesis of EV-G infection and for antiviral development. Traditional antiviral drug screening methods are time-consuming and labor-intensive, mainly relying on CPE assessment and plaque reduction assays. It is vital to focus research efforts on the development of a validated and rapid screening assay system in the search for potential antiviral compounds [29]. High-throughput screening systems developed based on viruses carrying reporter genes enable rapid and sensitive detection [20, 30].

Reverse genetics serves as a core methodology for elucidating resistance mechanisms and refining antiviral therapies. Iketani et al. employed a reverse genetics system to construct recombinant SARS-CoV-2 viruses harboring specific mutations, assessed their drug resistance and replicative capacity, and analyzed the spatial relationships between mutation sites and drug binding [31]. When designing recombinant reporter viruses for enteroviruses using reverse genetics system, two critical factors must be considered: the choice of insertion site and the selection of the reporter gene.

The enteroviral genome can be roughly divided into three regions: the region responsible for structural protein expression, the nonstructural protein region, and the untranslated regions (UTRs) located at both ends of the genome. Inserting foreign genes into the region encoding structural proteins may interfere with viral assembly and receptor binding, consequently affecting viral yield and infectivity. On the other hand, while insertion of foreign genes within the UTRs does not directly impact the synthesis and function of viral proteins, it can still alter the conformation of nearby elements involved in translation and replication, potentially resulting in replication deficiencies in the viral genome [32]. The non-structural protein region is a common site of recombination in Picornavirus, particularly at the 2 A/2B and 2C/3A junctions [33]. Notably, EV-G recombined with the PLP gene from torovirus at the 2C/3A region, confirming the recombination potential of this site and sparking our interest in the accommodation capacity of the EV-G 2C/3A region.

Regarding the selection of the reporter gene, theoretically, the smaller the foreign gene insertion, the lesser the impact on the virus. Choosing smaller-sized reporter genes while ensuring functionality is a key strategy for optimizing recombinant virus design and minimizing effects on viral replication and spread. Based on these considerations, we selected GFP(666 bp), RFP(717 bp), and iLOV(339 bp) as reporter genes to enable effective monitoring of viral infection and spread without significantly affecting the biological characteristics of the virus. The iLOV reporter gene (339 bp) is a truncated, photostable flavin-based fluorescent protein derived from plant phototropins [34]. It exhibits excitation/emission maxima at 446/485 nm, requires no exogenous cofactors, and is significantly smaller than GFP (666 bp) or RFP (717 bp), making it ideal for stable integration into viral genomes. These characteristics minimize metabolic burden and enhance genetic stability during serial passaging.

In this study, a recombinant infectious cDNA clone of EV-G-PLP was constructed using a reverse genetics system. The results of IFAs demonstrate that the infectious EV-G virus can be rescued by transfection of the recombinant plasmid into Marc-145 cells. Furthermore, in vitro growth kinetics and plaque assay also revealed that recombinant EV-G viruses exhibit similar proliferation rates and plaque morphology to the parental viruses. By replacing the PLP gene with a reporter gene and rescuing the virus, we observed that the r20GXNN-iLOV recombinant virus could be rescued and maintained genetic stability over ten generations. Although the r20GXNN-RFP and r20GXNN-GFP could also be rescued, their reporter genes were lost during passaging. This indicates that while the 2C/3A region of the 20GXNN strain can accommodate foreign genes, excessively long gene segments may lead to instability and loss. This contrasts with our previous conclusions on the infectious clone of the non-recombinant EV-G virus 17GXQZ [22], suggesting variability in different strains’ capacity to accommodate foreign genes at the 2C/3A region. This variability might explain why only certain strains undergo recombination clinically, as some may lack the capacity to support PLP gene recombination and therefore cannot produce recombinant viruses.

We excluded the r20GXNN-iLOV virus because its fluorescence was weak and quickly quenched fluorescence under microscopic observation, making it ineffective for screening purposes. In contrast, r20GXNN-RFP and r20GXNN-GFP showed strong, clear fluorescence signals under a microscope, allowing for a more accurate observation of viral proliferation. To avoid impacting experimental results, we used the P1 generation of r20GXNN-GFP virus that did not exhibit gene loss. We evaluated six drugs: ribavirin, salinomycin, niclosamide, chloroquine phosphate, Melaleuca Walternifolia essential oil, and chitosan, all of which have demonstrated antiviral activity against other viruses [35–39]. Ribavirin, salinomycin, niclosamide, and chloroquine phosphate demonstrated antiviral activity at concentrations of 400 µM, 2 µM, 0.02 µM, and 12 µM, respectively. However, Melaleuca alternifolia essential oil and chitosan did not show inhibitory effects on EV-G in this test. This lack of activity may be due to the drugs’ ineffectiveness against EV-G, and due to the low safe concentration levels in Marc-145 cells, which may have prevented the drug’s molecular weight from reaching the threshold required for antiviral effects.

In this study, the r20GXNN-iLOV demonstrated genetic stability during serial passaging. However, its utility in high-throughput drug screening was compromised by the low fluorescence intensity and rapid quenching characteristics of the iLOV tag. Specifically, weak fluorescent signals may lead to: (1) false-negative results during low viral load or early infection stages; (2) challenges in achieving long-term live-cell imaging, thereby hindering precise analysis of viral replication cycles. While smaller tags exert minimal interference on viral replication, they typically sacrifice optical performance, fundamentally reflecting the intrinsic conflict between the physicochemical properties of fluorescent proteins and genomic insertion tolerance. Subsequent construction of r20GXNN-GFP/RFP strains using larger GFP/RFP tags showed enhanced luminescence intensity and significantly improved photostability compared to iLOV. However, these strains exhibited reduced genetic stability relative to r20GXNN-iLOV, suggesting that oversized exogenous genes might exceed the physical packaging limit of the virus, manifesting as a negative correlation between insert length and viral packaging efficiency. The current viral vector platform demonstrates a rigid upper limit for exogenous gene carrying capacity, constraining the development of “high-signal/high-stability” dual-optimized viral strains. Recent advances in fluorescent protein engineering provide novel strategies to resolve this “intensity-stability” paradox. For instance, incorporation of the Q489K point mutation in iLOV can moderately enhance fluorescence intensity [40]. This modification may enable r20GXNN-iLOV to maintain sufficient fluorescence intensity. In contrast, the NanoLuc (NLuc) luciferase system addresses this challenge by splitting the luminescent core into the HiBiT peptide (11 amino acids) and the large LgBiT subunit. The HiBiT tag exhibits strong affinity for LgBiT, enabling NLuc reconstitution and subsequent bright luminescence while achieving synergistic advantages of ultracompact tag size and superior signal intensity [41]. Notably, Yu et al. successfully engineered an EV-A71 strain with wild-type-comparable genetic stability (> 10 passages) by inserting the HiBiT tag into the 5’UTR region of the viral genome [32]. Crucially, HiBiT-LgBiT complementation restored full NLuc activity in LgBiT-expressing host cells, a strategy with significant implications for this study.

In this study, we constructed an enterovirus G (EV-G) vector expressing various reporter genes to facilitate antiviral drug screening assays. The use of reporter genes, such as GFP, RFP, and iLOV, enabled us to monitor viral replication and assess the efficacy of antiviral compounds in real time. By optimizing the insertion sites within the viral genome, we ensured that the reporter genes did not significantly impact viral replication or pathogenicity. Our findings demonstrate that the EV-G vector can serve as a utilizable tool for high-throughput screening of antiviral drugs. Furthermore, the reporter gene signal correlated well with traditional viral replication assays, validating the reliability of our system. The development of this EV-G vector offers significant advantages for antiviral research. It allows for rapid and quantitative assessment of antiviral activity, reducing the time and resources required for traditional plaque assays. Additionally, we confirmed that the 2C/3A position of the 20GXNN virus can accommodate foreign genes, allowing the expression of other viral immunogenic proteins and highlighting its potential as a multivalent viral vector vaccine.

## Conclusions

In summary, this study successfully developed an EV-G vector expressing reporter genes, enabling a rapid, high-throughput platform for antiviral screening. The recombinant viruses retained infectivity and replication kinetics similar to the parental strain, validating their utility for drug efficacy assessment. Notably, r20GXNN-GFP provided a robust and stable marker for in vitro screening, with four compounds showing antiviral activity. This platform provides an insight and a tool to advance EV-G research and antiviral drug development.

## Supplementary Information

Below is the link to the electronic supplementary material.

Supplementary Material 1

## Data Availability

The datasets used and/or analysed during the current study are available from the corresponding author on reasonable request.
