# Supplementary figures and images for: Construction of enterovirus G expressing reporter genes for antiviral drug screening assays
Source: BMC Vet Res. 2025 Aug 13;21:515. doi: 10.1186/s12917-025-04960-0 (PMC12345017; doi:10.1186/s12917-025-04960-0)

Fig.2-D Original, unprocessed version of the image


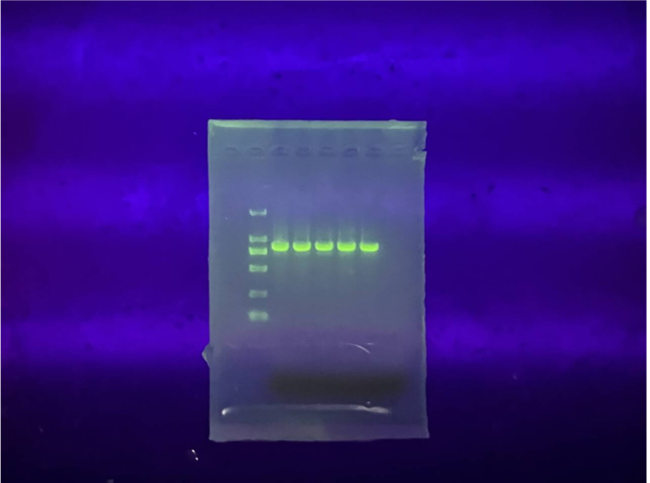

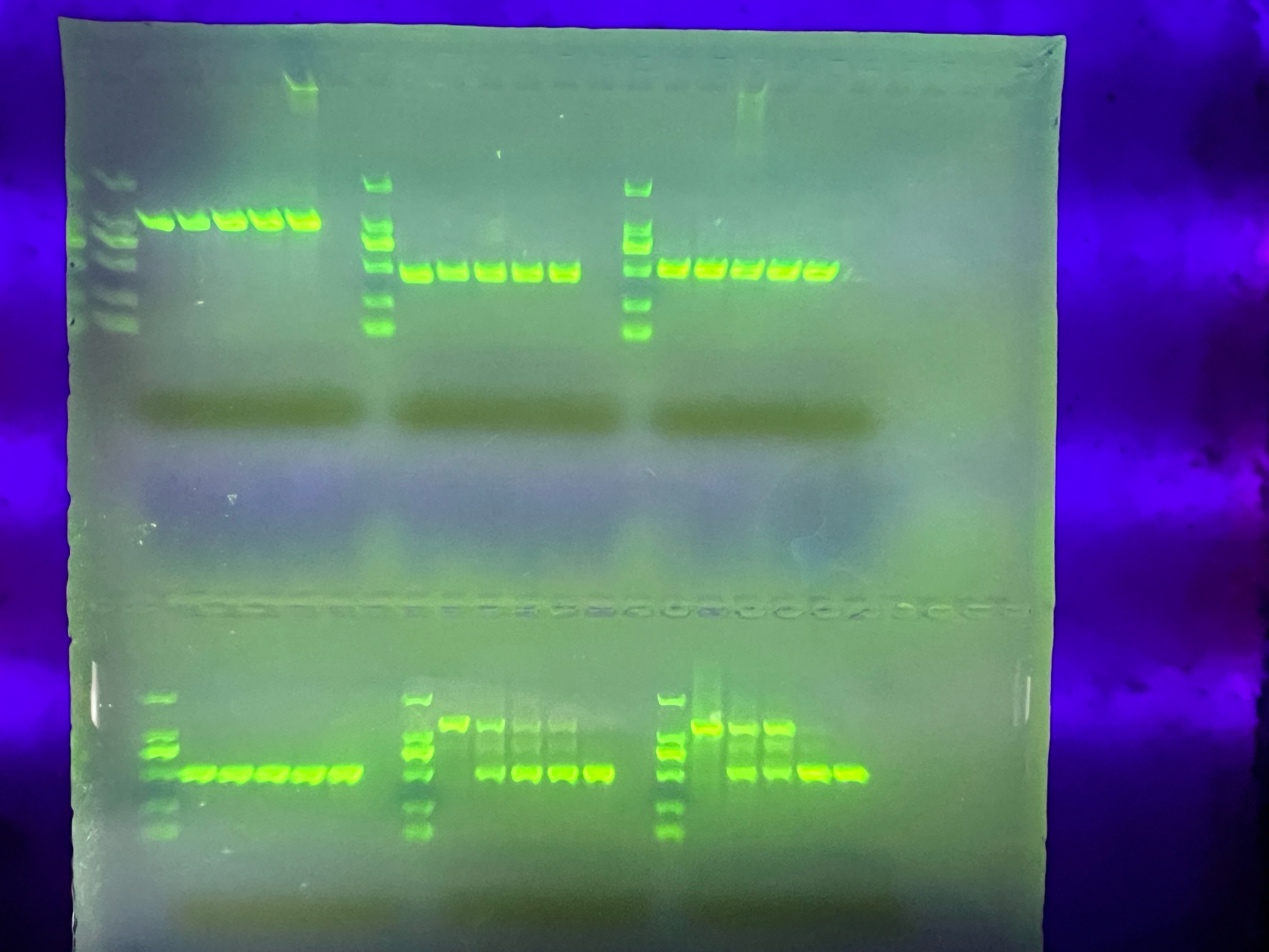

Supplement: Supplementary file 1 — Supplementary Material 1 [file 12917_2025_4960_MOESM1_ESM.docx]
